# Impact of coronary calcium patterns on procedural outcomes in CTO-PCI: a computed tomography-based multicenter study

**DOI:** 10.1007/s12928-025-01200-y

**Published:** 2025-10-11

**Authors:** Giuseppe Panuccio, Gerald S. Werner, Salvatore De Rosa, Daniele Torella, Yasuhiro Ichibori, Nicole Carabetta, Carsten Skurk, Patrick T. Siegrist, David M. Leistner, Ömer Göktekin, Kambis Mashayekhi, Ulf Landmesser, Youssef S. Abdelwahed

**Affiliations:** 1 Department of Experimental and Clinical Medicine, Magna Graecia University, 88100 Catanzaro, Italy; 2 Department of Cardiology, Angiology and Intensive Care Medicine, Deutsches Herzzentrum der Charite, Berlin, Germany; 3 Department of Cardiology, Heart Center, Goethe University Frankfurt, University Hospital, Frankfurt, Germany; 4 Department of Medical and Surgical Sciences, Magna Graecia University, Catanzaro, Italy; 5 Osaka Police Hospital, Osaka, Japan; 6 DZHK (German Centre for Cardiovascular Research), Berlin, Germany; 7 HerzZentrum Hirslanden Zurich, Zurich, Switzerland; 8 DZHK Partner Site Rhine-Main, Frankfurt, Germany; 9 Cardio-Pulmonary Institute, Partner Site Frankfurt, Frankfurt Am Main, Germany; 10 Memorial Bahcelievler Hospital, Istanbul, Turkey; 11 Heart Center Lahr, Internal Medicine and Cardiology, Lahr, Germany; 12 Berlin Institute of Health (BIH), Berlin, Germany

**Keywords:** Chronic total occlusions, Computed tomography, Coronary artery disease, Coronary imaging, Calcification, Precision medicine

## Abstract

Coronary calcium significantly increases complexity in chronic total occlusion percutaneous coronary intervention (CTO-PCI). Coronary computed tomography angiography (CCTA) enables precise CTO assessment. However, no prior study has proposed a CCTA–based morphological classification of calcium patterns and assessed its procedural impact. To propose and validate a novel seven-point CCTA-derived classification of calcium morphology, ranging from “spot” (≤ 10% cross-sectional area, CSA) to “full moon” (100% CSA). We retrospectively included 167 patients undergoing CTO-PCI with prior CCTA. The primary endpoint was procedural failure. Secondary endpoints included coronary perforations, procedural and fluoroscopic time, and number of guidewires and balloons. A progressive, stepwise increase in procedural failure (from 6.2% in spot to 26.7% in full moon lesions; *p* = 0.007) and coronary perforation rates (from 3.1% in spot to 13.3% in full moon lesions; *p* = 0.03) was observed across the seven identified calcium patterns. In multivariable analysis, calcium severity was independently associated with procedural failure (OR 1.2 per step; 95% CI 1.01–1.52; *p* = 0.04). Increasing calcium severity was also independently associated with procedural time (*B* =  + 4.7 min/step; *p* = 0.03), fluoroscopic time (*B* =  + 2.2 min/step; *p* = 0.04), number of guidewires (*B* =  + 0.30/step; *p* = 0.03) and balloons (*B* =  + 0.31/step; *p* = 0.005). Full-scale progression from “spot” to “full moon” corresponded to + 33 min increase in procedural and + 14 min in fluoroscopic time. A novel CCTA-based calcium classification showed a strong, independent association with procedural failure and complexity. Its reproducibility and integration potential make it a valuable tool for enhancing planning and safety in CTO-PCI.

**Graphical abstract:**

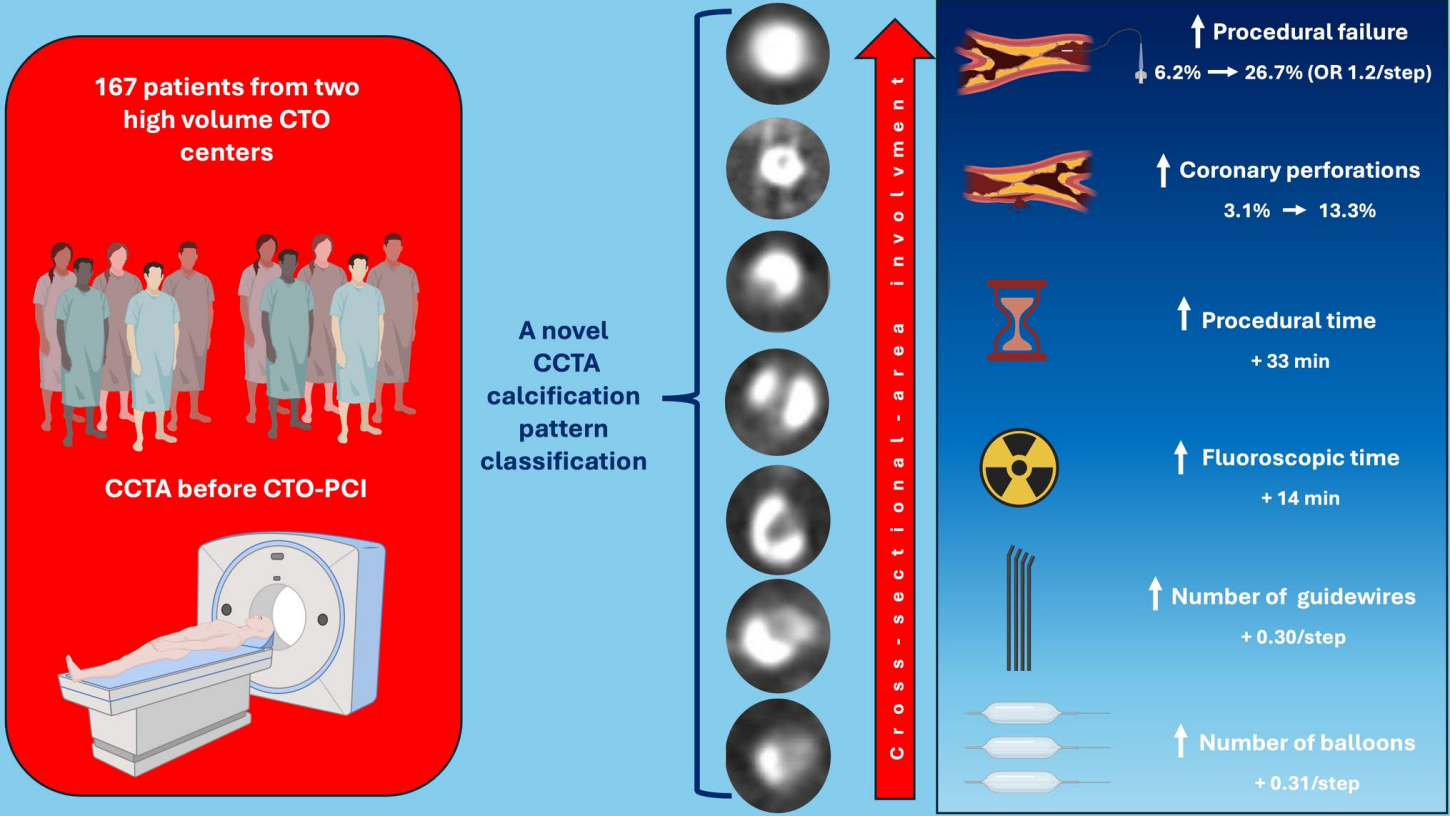

## Introduction

Coronary artery disease (CAD) is the third major cause of morbidity and mortality worldwide [1]. Coronary revascularization represents a cornerstone treatment in CAD management, significantly impacting short- and long-term outcomes [2, 3]. Chronic total occlusion (CTO) percutaneous coronary intervention (PCI) remains one of the most challenging procedures in interventional cardiology, with success rates still below those of non-CTO lesions despite major advances in techniques and devices [4–8]. One of the most challenging scenarios in CTO-PCI is heavy calcification (HC), which may significantly impact CTO-PCI procedures [9]. Specifically, HC may impede wire crossing, device delivery, and optimal stent expansion, potentially increasing the risk of complications such as perforation, restenosis, and stent thrombosis [10, 11]. Coronary computed tomography angiography (CCTA) has emerged as a valuable, non-invasive tool to evaluate CTO anatomy pre-procedurally [12–14]. While traditional angiography-based scoring systems offer only indirect assessment of calcification, CCTA provides a three-dimensional view of lesion characteristics, including calcification burden and distribution across the cross-sectional area (CSA) [15–17]. Particularly, CT-derived CTO scores such as CT-RECTOR and KCCT have shown that a calcium burden exceeding 50% of the CSA increases procedural complexity, underscoring the importance of CCTA in risk stratification and procedural planning [18, 19]. However, no prior study has proposed a detailed morphological classification of calcium patterns within CTO lesions using CCTA, nor has it correlated such patterns with procedural and clinical outcomes. Therefore, this study aims to propose a novel CCTA-based classification of calcification patterns in CTOs and to evaluate its predictive value on procedural success and complications in CTO-PCI.

## Methods

This was a retrospective, two-center observational study including 167 patients undergoing elective CTO-PCI and upstream preprocedural CCTA in 2 high-volume European centers (Deutsches Herzzentrum der Charité, Berlin, Germany, and Darmstadt Hospital, Darmstadt, Germany). Approval for the study protocol was obtained from the institutional review boards of both centers. The inclusion criteria comprised consecutive patients with severe CAD demonstrating evidence of at least one CTO, defined as Thrombolysis in Myocardial Infarction (TIMI) flow grade 0 with at least 3 months or unknown duration. Eligible patients undergoing CTO-PCI suffered from typical angina and underwent assessments for myocardial viability through functional tests. CTO-PCI procedures were executed according to the current European Society of Cardiology (ESC) guidelines [20]. Patients were excluded from the study if they suffered from myocardial infarction (MI) within 48 h, cardiogenic shock, or if the CTO-PCI was performed on a bypass graft. Finally, patients with suboptimal quality of the CCTA images and patients with incomplete clinical and procedural data were also excluded. Moreover, since the heterogeneity and the distinct procedural characteristics of in-stent CTOs, a sensitivity analysis was performed by excluding these patients. According to coronary angiography, J-CTO and EuroCTO (CASTLE) scores were assessed.

### CCTA acquisition and analyses

CCTA was conducted within 6 weeks before CTO-PCI. Every scan was carried out during end-diastole using an ECG-synchronized technique. To reduce artifact interference and to obtain high-detailed images, the smooth and the sharp Kernel (FC-14) was used for reconstruction. The specialized CT analysis software “Synapse Vincent system” (Fuji Film, Tokyo, Japan) was employed to evaluate every image. To evaluate the impact of calcium morphologies, only CTO lesions with visible calcification on CCTA were included. Using a specialized workstation (Synapse 3D, Fujifilm), images were analyzed offline by two experienced, blinded readers (G.P., Y. S.A.). The thin slab maximum intensity projection (MIP) and curved and stretched MPR were used to evaluate the anatomy of the CTO-vessel. The vessel course and the coronary angiography views were simulated using three-dimensional volume rendering images. CTOs were identified as segments without any luminal contrast enhancement. To measure vessel diameters and calcium extension, cross-sectional images of the CTO plaque were also taken at intervals no greater than 1.0 mm. Along the vessel’s axis, the CTO length was measured. The proximal cap features were classified as tapered or blunt. An angulation > 45° in the CTO segment was defined as a bending. Based on CCTA image interpretation, the KCCT and CT-Rector scores were calculated.

### Calcium patterns identification and classification

**Fig. 1:**
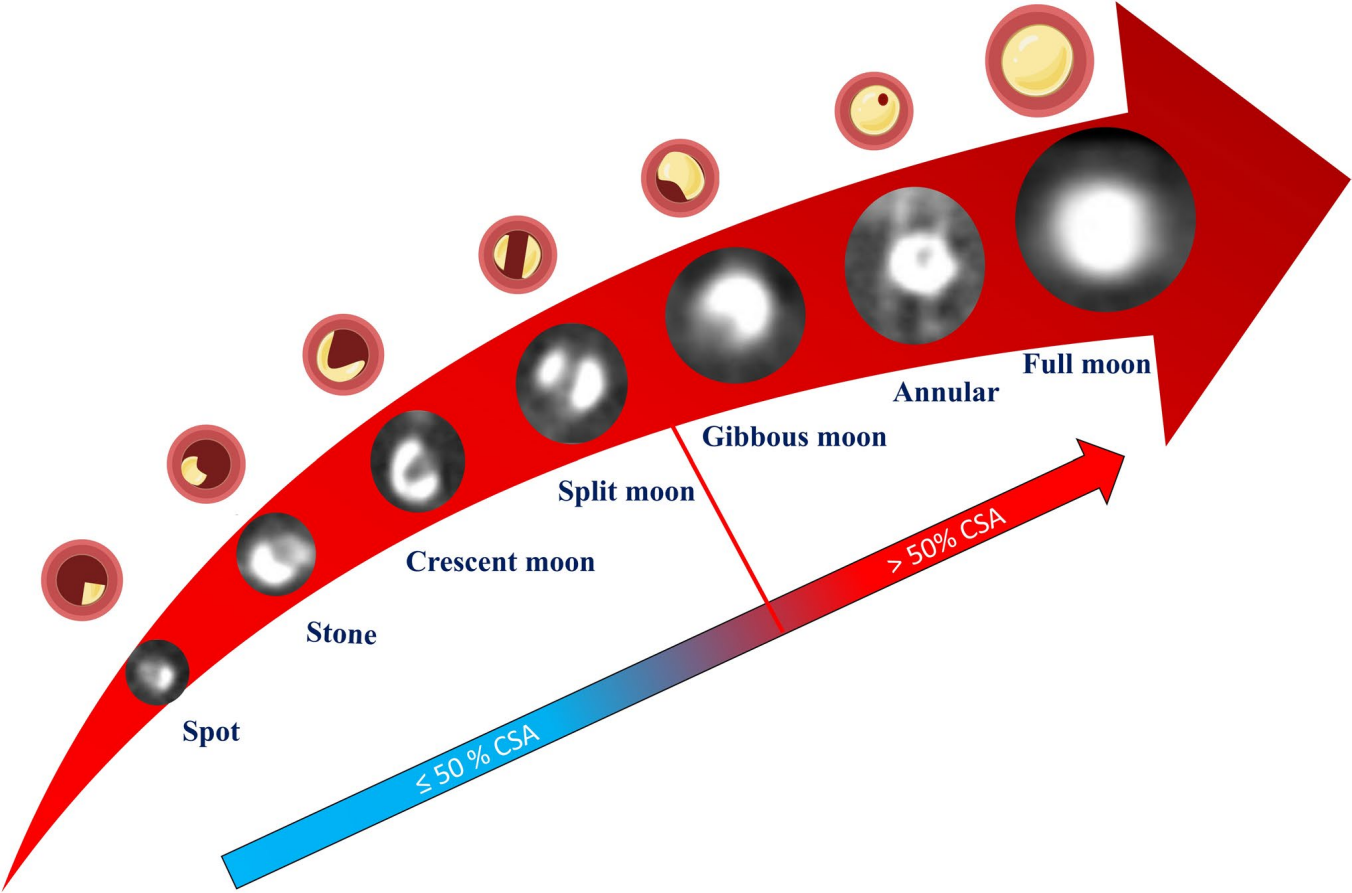
Seven-point CCTA-based classification of calcium patterns observed in CTOs

The calcifications were evaluated in a cross-sectional view to mitigate blurring artifacts, focusing on the segment with the highest calcification burden using an optimal widened image. Accordingly, we identified seven calcification patterns identifiable by CCTA, with a progressive extension across the CSA. The seven morphologies were derived from visual analysis of preprocedural CCTA in several CTO cases, selecting the most observed patterns. Definitions were standardized based on the arc and symmetry of calcium involvement in the vessel cross-section. Our novel proposed classification begins with the lowest grade, referred to as “spot.” This grade is characterized by a calcification that affects 10% or less of the cross-sectional area (CSA). The highest grade is called “full moon” lesion, which features a central 360° calcification that completely occludes the vessel, resulting in a CSA of 100% [21] (see Fig. 1 and Table 1). Lesions were also classified as having a high calcium burden if they exceed 50% of CSA. This dichotomization was performed to allow clearer evaluation of baseline characteristics and outcomes and to facilitate alignment with previous CCTA-based scoring systems, such as KCCT and CT-RECTOR, which used similar thresholds for calcium severity. Additional analyses according to low and high calcium burden are provided in supplementary data.

**Table 1:** Cross-sectional and morphological description of the seven-point CCTA-based classification of calcium patterns observed in CTOs

Pattern	% CSA at worst cross-section	Morphological criteria
Spot	0–10%	Small isolated focus of calcium
Stone	> 10–20%	Short contiguous arc of calcium
Crescent moon	> 20–30%	Single arc of calcium, < 180°
Split moon	> 30–50%	Two or more non-contiguous arcs
Gibbous moon	> 50–70%	Large continuous arc of calcium (180–250°)
Annular	> 70– < 100%	Nearly circumferential calcium ring
Full moon	100%	Complete 360° circumferential calcium

### Study endpoints

Experienced CTO operators performed all PCI procedures following European CTO Club strategies and standards [22]. Clinical and procedural data were collected prospectively or abstracted from records. The primary endpoint was procedural technical failure (defined as failure to recanalize the vessel with TIMI 3 flow). Secondary clinical outcomes included coronary perforations, in-hospital death, myocardial infarction (MI), and in-hospital major adverse cardiac events (MACE, defined as in-hospital death, MI, and clinically driven target vessel revascularization) [23]. Procedural outcomes included procedural (defined as time from administering local anesthesia to inserting the hemostasis device) and fluoroscopic time, contrast volume, as well as number of guidewires and balloons used.

### Statistical analysis

If continuous variables were normally distributed, they were expressed as mean ± standard deviation or median (interquartile range). We used *Q*–*Q* (quantile–quantile) plots and the Shapiro–Wilk test to determine the distribution of the continuous data. Consequently, the Student’s *t* test or the Mann–Whitney *U* test was used, as appropriate, to compare continuous data. Categorical variables were expressed by percentages and frequencies. Frequency differences were assessed using the Fisher exact test or Pearson’s chi-square test. Intraclass correlation coefficient (ICC) based on a two-way mixed-effects model was used to measure the reproducibility and inter-observer agreement of calcium quantification. Logistic regression analysis was performed to assess predictors of procedural failure. Variables with *p* value ≤ 0.20 in univariable analysis, or those with established clinical relevance, were included in the multivariable model. Pearson’s point biserial correlation was performed to assess collinearity between covariates. The primary exposure variable was calcium morphology, according to our novel seven-point classification, and was entered as an ordinal variable in multivariable logistic and linear regression analyses, in order to calculate the effect of every single calcium pattern in CTO-PCI. The effect size was quantified using Odds Ratios (OR) with 95% CI. Linear regression models were performed to evaluate the association between calcium patterns and procedural outcomes. Statistical significance was set at *p* < 0.05. Analyses were conducted using the Statistical Package for the Social Sciences (SPSS), version 25 (IBM Corp., Armonk, NY, USA).

## Results

A total of 167 patients were included. Table 1 describes the clinical and angiographic baseline features and the CTO complexity score (angiography and CT-based) calculations. Median age was 63.8 years (57.0–71.7), and 85.6% of the patients were men. A high cardiovascular risk profile was shown in most of the patients, including diabetes (29.3%), prior myocardial infarction (31.1%), prior PCI (37.1%), and prior CABG (35.9%). The most common target vessel for CTO-PCI was the right coronary artery (66.5%). Patients with high calcium burden morphologies were significantly older (*p* = 0.03) and presented with a higher incidence of diabetes (*p* = 0.01) and prior CABG (*p* < 0.001).

### Calcium pattern identification and re-classification

**Fig. 2:**
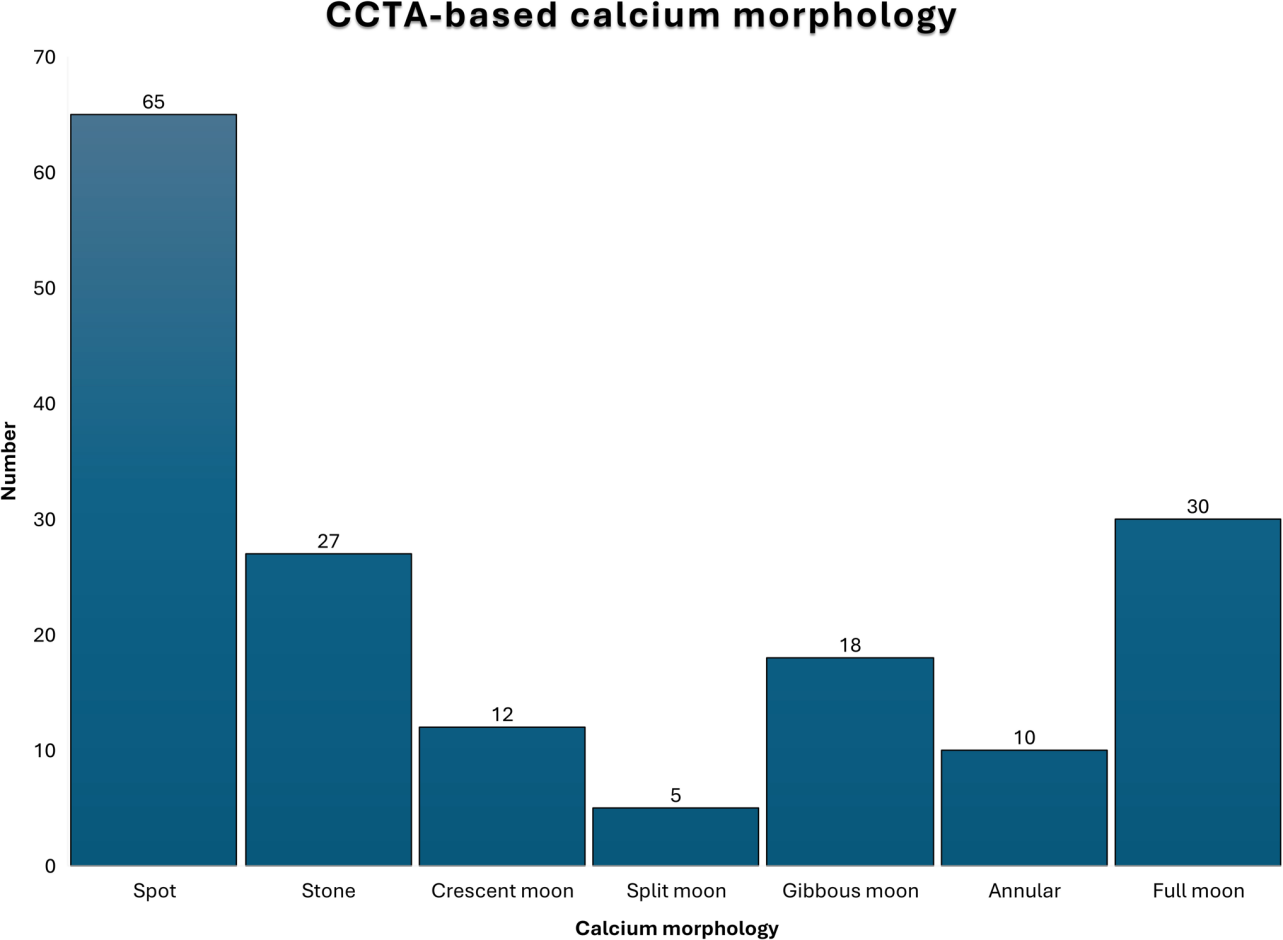
Calcium patterns distribution across the study population (*n* = 167)

Calcium morphologies were identified and classified according to our novel classification (Fig. 2). Interobserver agreement for calcium morphology classification was high (ICC 0.80; 95% CI 0.73–0.85; *p* < 0.001; Supplementary Fig. 1).

### Procedural outcomes

Procedural success was obtained in 143 patients (85.6%). Procedural failure occurred in 24 (14.4%) patients. The most frequent causes of procedural failure were the inability to cross the lesion with guidewires or to deliver balloons and coronary perforations. There was one in-hospital death. MACE occurred in 3 (1.8%) patients. Also, 11 patients experienced coronary perforation (6.6%), mostly involving large vessels and located at or near the most calcified segment. A detailed comparison of clinical and procedural outcomes is shown in Table 2.

**Table 2:** Baseline characteristics of the study population

Baseline characteristics	All *N* = 167	Spot *N* = 65	Stone *N* = 27	Crescent moon *N* = 12	Split moon *N* = 5	Gibbous moon *N* = 18	Annular *N* = 10	Full moon *N* = 30	*P* value
Age	63.6 ± 10.3	60.2 ± 9.8	65.49 ± 10.4	61.6 ± 14.8	58,84 ± 7.9	65.4 ± 9.68	65.5 ± 8.8	69.2 ± 7.6	**0.03**
Male sex	143 (85.6)	54 (83.1)	23 (85.2)	12 (100)	3 (60)	16 (88.9)	8 (80)	27 (90)	0.52
Family history of CAD	44 (26.3)	18 (27)	7 (25.9)	5 (41)	2 (4)	3 (16.6)	2 (2)	7 (23)	0.44
Hypertension	136 (81.4)	52 (80)	24 (88)	8 (66)	4 (80)	16 (88)	6 (60)	26 (86)	0.89
Dyslipidemia	123 (73.6)	46 (70)	17 (62.9)	9 (75)	3 (60)	14 (77)	7 (70)	27 (90)	0.11
Diabetes	49 (29.3)	17 (11)	6 (22)	1 (8)	1 (20)	7 (38)	3 (30)	14 (46.6)	**0.03**
Smoking	85 (50.8)	35 (53)	13 (48)	9 (75)	2 (40)	9 (50)	6 (60)	11 (36)	0.57
Peripheral artery disease (PAD)	19 (11.3)	5 (7)	4 (14)	2 (16)	1 (20)	1 (5)	1 (10)	5 (16)	0.42
COPD	6 (3.5)	2 (3)	0	0	0	2 (11)	0	2 (6)	0.24
Chronic kidney disease (CKD)	11 (6.5)	2 (3)	3 (11)	0	0	2 (11)	0	4 (13)	0.15
Dialysis	3 (1.7)	0 (0)	1 (3.7)	0 (0)	0 (0)	1 (5.5)	0	1 (3.3)	0.23
Prior stroke	8 (4.7)	3 (4)	2 (7)	0	1 (20)	1 (5)	0	1 (3)	0.67
Previous MI	52 (31.1)	22 (33.8)	7 (25.9)	5 (41.6)	1 (20)	8 (44.4)	2 (20)	7 (23.3)	0.05
Previous PCI	62 (37.1)	19 (29.2)	8 (29.6)	6 (50)	1 (20)	11 (61.1)	4 (40)	13 (43.3)	0.05
LVEF	54.0 [40.0–57.5]	51.6 [41.5–59]	45.6 [30–57.5]	50 [40–55]	57.5 [52–62]	57.5[53–61]	38 [35–42]	49.5[45–52]	0.7
Prior CABG	60 (35.9)	14 (21.5)	9 (33.3)	1 (8)	0	6 (33.3)	5 (50)	25 (83.3)	** < 0.01**
Right coronary artery	111 (66.4)	43 (66.2)	17(63.0)	6 (50.0)	3 (60.0)	13 (73.2)	7 (70.0)	22 (73.3)	0.85
Left anterior descending artery	27 (16.1)	8 (12.3)	6 (22.2)	3 (25.0)	1(20.0)	4 (22.2)	0 (0)	5 (16.7)	0.59
Left circumflex	29 (17.3)	14 (21.5)	4 (14.8)	3 (25.0)	1 (20.0)	1 (5.6)	3 (16.6)	3 (10)	0.68
CTO characteristics									
CTO location									
Ostial	33 (19.8)	6 (9)	7 (25)	2 (16)	1 (20)	6 (33.3)	4 (40)	7 (23.3)	0.11
Proximal	93 (55.7)	40 (61.5)	16 (59.2)	8 (66.6)	3 (60)	6 (33.3)	5 (50)	15 (50)	0.44
Mid	35 (20.9)	17 (26.1)	4 (14.8)	1 (8)	1 (20)	5 (27.7)	0	7 (23.3)	0.41
Distal	6 (3.6)	2 (3)	0	1 (8)	0	1 (5.5)	1 (10)	1 (3.3)	0.75
In-stent CTO	19 (11.4)	2 (3)	4 (14.8)	1 (8)	0	2 (11.1)	6 (60)	4 (13.3)	** < 0.001**
Bifurcation involvement	50 (29.9)	20 (30.7)	7 (25)	2 (16)	2 (40)	3 (16.6)	2 (20)	14 (46.6)	0.28
Stump morphology	90 (53.8)	33 (50.7)	16 (59.2)	8 (66.6)	3 (60)	10 (55.5)	7 (70)	13 (43.3)	0.65
Calcification	113 (67.6)	34 (52.3)	16 (59.2)	6 (50.0)	5 (100)	15 (83.3)	7 (70)	30 (100)	** < 0.001**
Previous attempts	52 (31.3)	16 (24.6)	10 (37)	4 (33.3)	2 (40)	10 (55.5)	1 (10)	9 (30)	0.76
Radial access	113 (67.6)	42 (64.6)	17 (62.9)	6 (50)	3 (60)	15 (83.3)	7 (70)	23 (76.6)	0.49
Contralateral injection	108 (65.1)	42 (64.6)	17 (62.9)	7 (58.3)	3 (60)	13 (72.2)	6 (60)	20 (66.6)	0.98
IVUS	90 (53.8)	39 (60)	11 (40.7)	7 (58.3)	4 (80)	9 (50)	5 (50)	15 (50)	0.54
Antegrade recanalization	114 (68.3)	49 (75.3)	15 (55.5)	7 (58.3)	1 (20)	11 (61.1)	10 (100)	21 (70)	**0.02**
Antegrade dissection and re-entry	11 (6.5)	6 (9.2)	1 (3.7)	1 (8.3)	0	0	1 (10.0)	2 (6.7)	0.81
Parallel wire	27 (16.1)	11 (16.9)	7 (25)	1 (8)	0	2(11.1)	0	6 (20)	0.45
Retrograde recanalization	69 (41.3)	27 (41.5)	11 (40.7)	6 (50)	4 (80)	8 (44.4)	1 (10)	12 (40)	0.27
J-CTO score	2 (1–3)	2 (1–3)	3 (1.75–4)	2 (2–4)	2.5 (3–3.5)	2 (3–4)	2 (1–3.25)	3 (1–4)	**0.02**
EURO-CTO score	3 (2–5)	3 (1–4)	3 (2–5)	4(2–6)	4 (3–5)	3 (2–5)	3 (1.75–3.5)	4 (3–5.25)	**0.01**
KCCT score	3 (2–4)	3 (2–3)	2.5 (2–4)	3 (2–4)	3 (2–4.5)	4 (3–5)	3.5 (2.75–4.25)	5 (4–6)	** < 0.001**
CT-RECTOR score	2 (1–3)	2 (1–2)	2 (1–3)	2 (1–3)	2 (1–3)	3 (2–4)	2.5 (1.75–3)	3 (2–4)	** < 0.001**

The definition for the bold in tables is that the p value was statistically significant

**Fig. 3:**
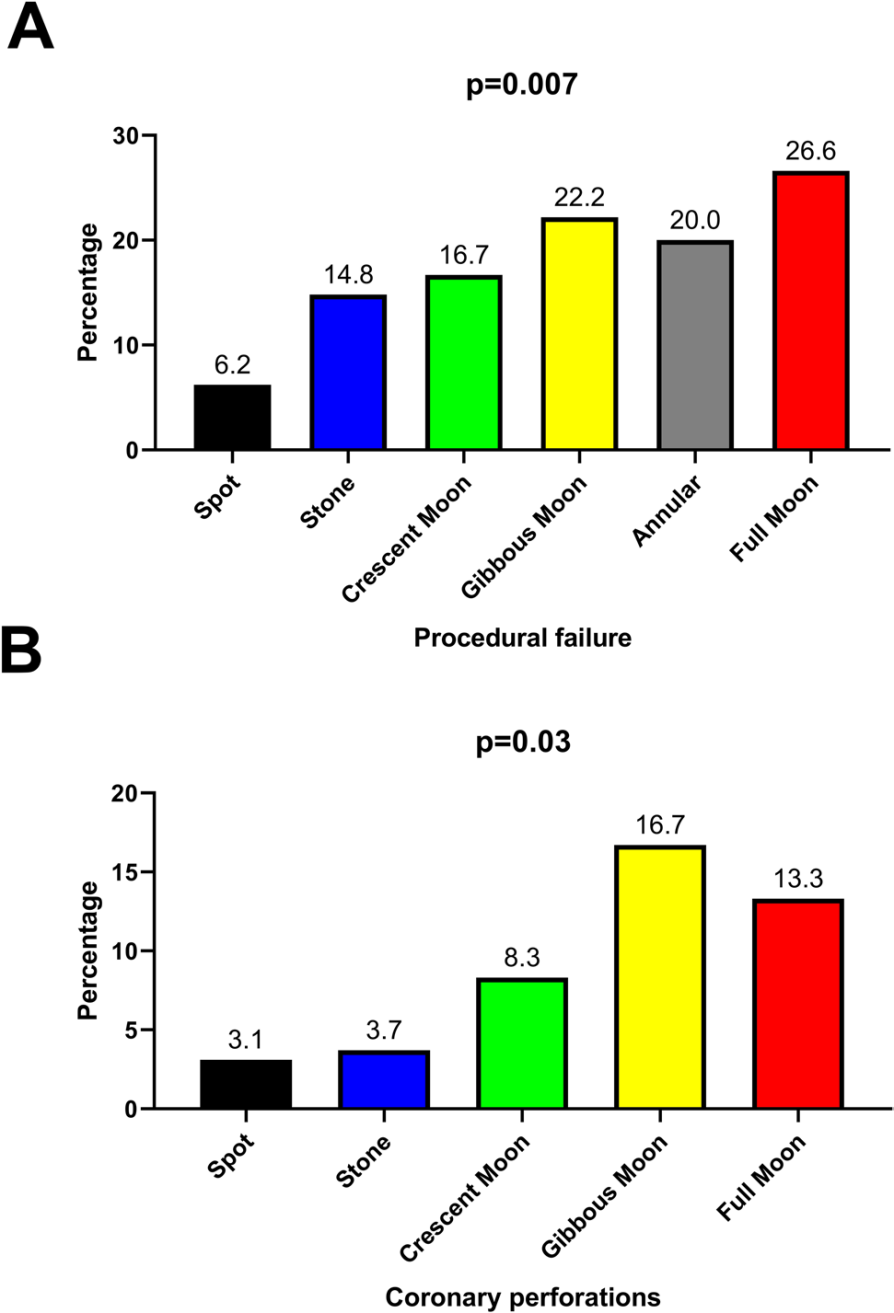
Procedural failure (**A**) and coronary perforations (**B**) incidence for single calcium morphology. Calcium morphologies with no events were not included in the figure

A progressive and significant increase in procedural failure was observed across the calcification patterns, ranging from 6.2% in “spot” morphology to 26.7% in “full moon” lesions (*p* for trend = 0.007; Table 2; Fig. 3A). Similarly, coronary perforation rates increased from 3.1% in “spot” to 13.3% in “full moon” lesions (*p* = 0.03; Fig. 3B). In multivariable logistic regression analysis, calcification pattern severity, according to our novel classification, was an independent predictor of procedural failure (OR 1.2 per step; 95% CI 1.01–1.52; *p* = 0.04; Table 3). Sensitivity analyses including each individual component of the J-CTO score yielded consistent results with our main findings, with calcification pattern severity remaining independently associated with procedural failure and complexity (Supplementary Table 3 and 4). In-stent CTOs were observed in 19 patients (11.3%). Since their distinct procedural characteristics and their heterogeneity, a sensitivity analysis was performed by excluding these patients from the primary analysis (supplementary Fig. 5), which yielded consistent results with the main analysis. In linear regression models adjusted for age, diabetes, and previous CABG, calcification pattern severity was independently associated with longer procedural time (B coefficient 4.7 min/step; 95% CI 0.2–9.1; *p* = 0.03), fluoroscopic time (B coefficient 2.2 min/step; 95% CI 0.02–4.3; *p* = 0.04), number of guidewires (B 0.3/step; 95% CI 0.02–0.62; *p* = 0.03) and balloons (0.31/step; 95% 0.09–0.52; *p* = 0.005), as described in Table 4. The full range from spot to full moon calcification (7-point scale) corresponded to an estimated cumulative increase of approximately + 33 min in procedural time, + 14 min in fluoroscopic time, + 1.8 guidewires, and + 1.9 balloons used (Table 5).

**Table 3:** Comparison of procedural and in-hospital outcomes for single calcium morphology

Outcome	All *N* = 167	Spot plaque *N* = 65	Stone plaque *N* = 27	Crescent moon plaque *N* = 12	Split moon plaque *N* = 5	Gibbous moon plaque *N* = 18	Annular plaque *N* = 10	Full moon plaque *N* = 30	*P* value
Procedural failure	24 (14.4)	4 (6.15)	4 (14.81)	2 (16.66)	0 (0)	4 (22.22)	2 (20)	8 (26.66)	**0.007**
In hospital death	1 (0.5)	0	0	0	0	0	1 (10)	0	0.59
MI	2 (1.1)	0	1 (3.7)	1 (8.3)	0	0	0	0	0.22
MACE	3 (1.8)	0	1 (3.7)	1 (8.3)	0	0	1 (10)	0	0.15
Coronary perforations	11 (6.6)	2 (3)	1 (3.7)	1 (8.3)	0	3 (16.6)	0	4 (13.33)	**0.03**
Vascular complication	3 (1.8)	0	1 (3.7)	1 (8.3)	0	0	0	1 (3.3)	0.6
Conversion to surgery	2 (1.1)	0	0	1 (8.3)	0	0	0	1 (3.3)	0.39
CIN	11 (6.5)	8 (12.3)	2 (7.4)	0	0	0	0	1 (3.3)	0.24
Intense debulking (rotational atherectomy/coronary lithotripsy)	17 (10.1)	1 (1.5)	0	0	1 (20)	3 (16.6)	1 (10)	11 (36.6)	** < 0.001**
Rotational atherectomy	6 (3.5)	0	0	0	0	0	0	6 (20)	** < 0.001**
Intravascular lithotripsy	11 (6.6)	0	0	0	1 (20)	2 (11.1)	1 (10)	7 (23.3)	** < 0.001**
Both	1 (0.5)	0	0	0	0	0	0	1	0.60
Intense debulking or inability to cross	35 (20.9)	4 (6.1)	2 (7.4)	1 (8.3)	1 (20)	7 (38.8)	3 (30)	17 (56.6)	** < 0.001**
Total number of guidewires	6.5 (4–9)	6 (4.25–8)	5.5 (3–9.25)	8.5 (5.5–11.5)	11 (6.5–13)	5 (3–9.5)	5.5 (2.75–9.75)	8.5 (5–11)	0.06
Total number of balloons	5 (3–6.5)	4 (3–5)	5 (3–7)	4 (2–6)	6 (4–6)	5.5 (4–8.75)	4 (2.75–6.25)	7 (4–10)	**0.03**
DES number	3 (2–4)	3 (2–4)	3 (2–4)	3 (2–4)	3 (2–4)	3 (2–3)	2.5 (1–4.75)	3.5 (3–4)	0.78
DES length	88 (60–108)	84.5 (54.5–104.25)	96 (76–115)	84 (65.5–103.75)	64 (53–113.5)	96 (58–109)	75 (17–115)	101 (65–112)	0.69
DES diameter	3.5 (3.5–4)	4 (3.31–4)	3.5 (3.25–4)	3.62 (3.37–4)	3.5 (3.25–4)	4 (3.5–4)	3.25 (2,43–4)	3.5 (3.5–4)	0.72
Procedural time	143 (105–176.2)	129.5 (91.25–159.75)	131 (103–175)	146 (118.5–182.5)	149 (114.5–229)	137.5(87.75–164)	140 (105–181)	172.5 (119–236.25)	**0.050**
Fluoroscopic time	42 (28.8–66.3)	37 (25–59.05)	36 (25.85–52.8)	53.15 (40.57–79.77)	49.9 (37.25–92.75)	37.1 (26.5–69.25)	40.55 (25.75–76.7)	65.15 (38.15–80)	**0.02**
Contrast volume (ml)	230 (180–280)	237.5 (180–297.25)	230 (172.75–338.75)	234.5 (182.5–295.75)	300 (240–385.5)	220 (172–251.75)	231 (200–269.25)	223.5 (169.75–251)	0.5

The definition for the bold in tables is that the p value was statistically significant

**Table 4:** Multivariable linear regression model for secondary procedural outcomes

Variable	B coefficient	95% CI	*p* value
Procedural time (min)			
Calcification pattern	4.7	0.2–9.1	**0.03**
Prior CABG	22.4	1.4–43.5	**0.03**
Age	− 0.3	− 1.3–0.53	0.40
Diabetes	− 16.2	− 36.4–4.0	0.11
Fluoroscopic time (min)			
Calcification pattern	2.2	0.02–4.3	**0.04**
Prior CABG	10.9	0.6–21.2	**0.03**
Age	− 0.2	− 0.7–0.1	0.25
Diabetes	− 6.0	− 15.9–3.9	0.23
Number of guidewires			
Calcification pattern	0.3	0.02–0.62	**0.03**
Prior CABG	1.0	− 0.4–2.4	0.15
Age	− 0.05	− 0.11–0.009	0.09
Diabetes	− 0.4	− 1.8–0.92	0.51
Number of balloons			
Calcification pattern	0.3	0.09–0.52	**0.005**
Prior CABG	0.6	− 0.4–1.6	0.24
Age	− 0.04	− 0.05–0.04	0.87
Diabetes	0.19	− 0.8–1.2	0.70

The definition for the bold in tables is that the p value was statistically significant

**Table 5:** Univariable and multivariable regression analysis for the primary endpoint

Variable	Univariable analysis	*p* value	Multivariable analysis	*p* value
Calcification pattern	1.2 (1.06–1.5)	**0.008**	1.2 (1.01–1.52)	**0.04**
Prior CABG	1.9 (0.8–4.7)	0.12	1.2 (0.4–3.3)	0.71
J-CTO score	1.2 (0.9–1.7)	0.17	1.1 (0.84–1.6)	0.33

The definition for the bold in tables is that the p value was statistically significant

## Discussion

In this multicenter study, we presented a novel CCTA-based morphological classification of coronary calcium in CTOs and demonstrated its association with procedural outcomes in CTO-PCI. We found that high calcium pattern morphologies according to our novel classification were independently associated with procedural failure. Additionally, increasing calcium severity was independently associated with longer procedural time, fluoroscopic time, higher number of guidewires and balloons used, underscoring procedural complexity. Previous scoring systems for CTO PCI, such as J-CTO and EURO CTO CASTLE scores, included calcium presence as a binary variable based on angiography [24, 25]. Notably, the J-CTO score considers the presence of any calcification based on angiography, without accounting for severity or morphology. In contrast, the CASTLE score includes only severe calcification. This key distinction is often overlooked in literature and highlights the need for a more nuanced, morphology-based classification. Our classification addresses this gap by stratifying calcium severity on a seven-point morphological scale using CCTA, thereby providing a more detailed and predictive assessment of CTO complexity. Moreover, our calcium classification has emerged as an independent predictor of procedural failure, even after adjustment for the J-CTO score, which incorporates key factors such as lesion length, CTO angle, and blunt stump. More recently, CCTA-derived scores like CT-RECTOR and KCCT have added calcium ≥ 50% of CSA to predict procedural failure. However, to our knowledge, no previous study has proposed a morphological classification of CTO calcium patterns based on CCTA and evaluated its correlation with procedural outcomes. Our classification allows for a more nuanced view of the lesion, offering an objective, reproducible, and preprocedural assessment of calcium morphology that reflects increasing procedural complexity. Unlike previous CCTA-derived scores that dichotomize calcium burden, our study provides a morphology-based classification that captures the full spectrum of calcium severity, revealing a progressive and dose-dependent relationship with procedural failure and complexity. This seven-point scale not only reflects the heterogeneity of calcium patterns in CTO lesions but also enables a quantifiable, progressive evaluation of their procedural impact. In order to evaluate its clinical implication, we performed a supplementary ROC curve analysis for the primary endpoint, showing that our calcification morphology classification had the highest predictive performance for procedural failure (AUC 0.662, *p* = 0.015), surpassing both a dichotomous classification of calcium burden > 50% (AUC 0.61, *p* = 0.085) and existing CTO scores (KCCT: AUC 0.60, *p* = 0.10 and CT Rector: AUC 0.54, *p* = 0.48, Supplementary Fig. 3). The optimal cut-off point was identified as the crescent moon pattern, indicating that the risk of procedural failure significantly increases from this morphology onward. Importantly, this demonstrates the incremental predictive value of our morphology-based approach over currently available CCTA-based score that dichotomize calcium assessment. It should also be acknowledged that proximal calcification is another well-recognized determinant of procedural complexity in CTO-PCI, including decreased wire manipulation and proximal cap visualization. While our classification focused on calcium morphology within the occluded segment, proximal calcification may also have an impact on procedural complexity in CTO-PCI. Our study aligns with recent evidence reporting the impact of prior CABG on procedural complexity in CTO-PCI [26, 27]. However, in our analysis, calcification type had a cumulative independent and larger impact than prior CABG on the primary outcome of procedural failure. Collateral circulation, as assessed by the Rentrop grading system, is an important determinant of procedural success in CTO-PCI [28, 29]. However, in our study, collateral grading was not included in the main analyses. Future works integrating both calcium morphology and collateral status may further refine procedural risk prediction. An important and clinically relevant observation of this study was the significantly higher incidence of coronary perforation in the most severely calcified morphologies, with the highest rate in the gibbous moon morphology (16.7%). This pattern is characterized by a pronouncedly eccentric calcium distribution, which may create asymmetric wall compliance. The non-calcified segment, acting as a zone of least resistance during balloon inflation or device expansion, is particularly vulnerable to focal rupture. These findings support the notion that not only the extent but also the geometry of calcium deposition influences the risk of procedural complications, reinforcing the value of morphology-based pre-procedural imaging assessment. Although calcified CTOs often display mixed morphologies along the occluded segment, for ensuring reproducibility and comparability of our findings, we classified lesions according to the most severe cross-sectional pattern observed. This approach is consistent with previous imaging-based classifications and ensures that the dominant feature driving procedural complexity is captured. Nevertheless, the higher incidence of coronary perforation in the gibbous moon morphology suggests that even when coexisting with other patterns, its presence may carry specific vulnerability. Future larger studies could explore multi-pattern coding to clarify whether the presence of gibbous moon, irrespective of dominance, represents an independent determinant of perforation risk. Our study included a minority of patients (11.3%) who presented with in-stent CTOs. These lesions represent a heterogeneous entity with distinct procedural characteristics compared to de novo CTOs. Their heterogeneity may result from either newly developed calcium within the stent or old calcium outside the stent. In our cohort, five patients (26.3%) apparently presented a newly developed in-stent calcium, while 14 patients (73.6%) presented calcification during the first stent implantation, which was not quantified by CCTA. Although the sensitivity analysis excluding in-stent CTOs showed consistent results with the main analysis, the variability and limited sample size of this subgroup did not allow our study to be adequately powered to assess meaningful differences between in-stent and de novo CTOs. Accordingly, our results should not be extrapolated to in-stent CTOs; rather, they underscore the need for dedicated studies specifically addressing this heterogeneous subgroup. Another key finding of our study is the clear, dose-dependent relationship between calcium morphology and multiple procedural outcomes. Each incremental step in our seven-point calcification severity scale was independently associated with a progressive and significant increase in procedural time, fluoroscopic time, number of guidewires, and balloons used. The clear dose–response relationship observed across increasing calcium severity pattern demonstrates that each morphological category contributes deeply to procedural complexity. The estimated increase in procedural and fluoroscopic time from the least to the most severe calcium type approached 33 and 14 min, respectively, underlining that a CCTA-based calcium morphology assessment is not merely descriptive but strongly predictive of technical complexity. The observed association between calcification patterns and both procedural and fluoroscopic times may also serve as a valid surrogate for increased guidewire manipulation time, which was not directly assessed in this study. By demonstrating the progressive association of calcium morphologies with procedural metrics, our findings validate the clinical relevance and the added value of this classification and suggest that calcium severity should not be considered a binary feature, but as a spectrum with quantifiable clinical impact. This categorization may be a reproducible and powerful tool to support preprocedural decision-making in CTO-PCI. It can assist case selection, strategy planning, and equipment readiness—for example, also predicting the need for plaque modification devices such as intravascular lithotripsy or rotational atherectomy [9, 21, 30], which use was significantly higher in patients with high calcium burden morphologies. Moreover, its visual nature and strong interobserver agreement make it suitable for broader clinical application and future integration into automated CCTA workflows. From a clinical standpoint, the proposed classification provides a simple and intuitive tool to guide procedural planning in CTO-PCI. Mild calcification patterns may be usually managed with standard approaches, while more severe calcium morphologies may prompt early use of support and plaque modification devices. Furthermore, eccentric patterns like the gibbous moon may warrant more cautious dilation to mitigate perforation risks. Unlike burden-based score, this morphology-driven approach offers actionable insight for real-time decision-making. Given the complexity associated with CTO-PCI, procedural safety remains a significant concern in contemporary practice [31]. By enabling a thorough pre-procedural evaluation of calcium morphology, CCTA may significantly aid in strategizing and reducing complications. In “precision medicine,” coronary imaging, especially CCTA, provides a comprehensive analysis of CTO plaques, which can predict complexity and enhance safety during CTO PCI.

## Limitations

Our study has some limitations. First, its retrospective nature and small sample size may limit the generalizability of the findings and hinder categorical modeling across all patterns. However, calcification patterns were analyzed as ordinal variables, which reflect the biological plausibility of a progressive dose–response relationship, since each morphology is characterized by an incremental increase in cross-sectional calcium burden. Although both centers were high-volume CTO-PCI centers, variations in operator experience and institutional strategies may have influenced the outcomes. Second, while the CCTA analysis focused on the cross-sectional segment with the greatest calcification burden, the longitudinal extent of calcification across the vessel was not systematically included in the classification. Additionally, the heterogeneity, the distinct procedural characteristics, and the limited sample size of in-stent CTOs limited our ability to assess differences with de-novo CTOs, and therefore, our findings should not be extrapolated to this population. Third, we acknowledge that lesions without calcification were excluded from the present analysis, which limits generalizability to non-calcified CTOs. However, this was intentional to specifically address the procedural impact of calcium morphology. Furthermore, our classification was based exclusively on the cross-sectional morphology of calcium, which we considered the most reproducible and clinically applicable. Other parameters such as longitudinal length of calcification and its radial thickness were not systematically evaluated in our dataset. Although these features may provide complementary information, their absence may have limited the comprehensiveness of our assessment. Future studies integrating both cross-sectional and longitudinal calcium metrics could further refine risk stratification in CTO-PCI. Fourth, while we examined various procedural metrics and in-hospital outcomes, we did not include a specific analysis of guidewire manipulation time or long-term clinical outcomes. Moreover, the use and availability of dedicated software for interpreting CCTA images vary, which limits its adoption in routine practice. Finally, although our classification demonstrated consistent associations with multiple procedural outcomes, it has not yet undergone external validation. Therefore, future studies are needed to assess this CCTA-based scoring system in independent cohorts and across different clinical settings.

## Conclusions

CCTA provides a thorough evaluation of CTO morphology and extent of calcification. A new morphological classification system, based on CCTA, categorizes calcium patterns in CTOs and demonstrates a clear, dose-dependent relationship with the complexity of PCI for CTOs. This classification is reproducible and can be easily incorporated into pre-procedural workflows. Adopting this system may help customize procedural strategies, enhance planning, and support more efficient and safer CTO-PCI procedures.

## Data Availability

This work was supported by grants from the Italian Ministry of University and Research (PNRR—National Center for Gene Therapy and Drugs based on RNA Technology No. CN00000041) and from the Italian Ministry of Health (POS4 ‘Cal-Hub-Ria’ No. T4-AN-09; PNRRMAD-2022-12376814.
